# Between panic and motivation: did the first wave of COVID-19 affect scientific publishing in Mediterranean countries?

**DOI:** 10.1007/s11192-022-04391-w

**Published:** 2022-06-07

**Authors:** Mona Farouk Ali

**Affiliations:** Department of Information Science, Faculty of Arts, Helwan University, Cairo, Egypt

**Keywords:** COVID-19, Scientometrics, Bibliometrics, Scientific publishing, Research analysis, Research performance, Mediterranean countries, Crisis

## Abstract

Due to the ongoing COVID-19 pandemic that began in the winter of 2020, all communities and activities globally have been positively or negatively affected. This scientometric study raises an interesting question concerning whether the volume and characteristics of scientific publishing in all disciplines in 23 Mediterranean countries have been impacted by the pandemic and whether variations in the cumulative totals of COVID-19 cases have resulted in significant changes in this context. The Scopus database and SciVal tool supplied the necessary data for the years targeted for comparison (2019 and 2020), and the annual growth rates and differences were computed. The study used the Mann–Whitney test to examine the significance of the differences between the two years and the Spearman and Kendall correlation tests to evaluate the effect of the number of infections on these differences for all aspects of scientific performance. The findings demonstrated that the COVID-19 pandemic served as a powerful incentive, and the Mediterranean region experienced considerable differences in the volume and features of publications during this crisis. The most substantial implications were the significant growth from 3.1 to 9.4% in productivity and the increases in the annual growth rates of international collaboration, by 12% for the collaboration among Mediterranean countries and 10% for collaboration with the top ten epidemic countries. It was also proven that some characteristics of the publications were positively correlated with the total number of infections. This investigation can help university leaders and decision-makers in higher education and research institutions in these countries make decisions and implement measures to bridge the gaps and motivate researchers in all fields to conduct more research during this ongoing pandemic.

**Supplementary Information:**

The online version contains supplementary material available at 10.1007/s11192-022-04391-w.

## Introduction

Since COVID-19 was declared a Public Health Emergency of International Concern (PHEIC) by the World Health Organization (WHO) on January 30, 2020 (World Health Organization, 2020b), diverse occupations and activities have been dramatically impacted globally, such as business organizations (Seetharaman, 2020), creative and cultural industries (Comunian & England, 2020), and self-employed workers (Beland et al., 2020). The active case count, physical distancing, isolation, workplace lockdowns, and transcendent family care have been highlighted as influences controlling workability and productivity during the COVID-19 pandemic (Craig & Churchill, 2020; Czymara et al., 2020; Dey et al., 2020; McIntyre & Lee, 2020; Truxillo et al., 2020). Several psychological studies have revealed high rates of generalized anxiety, depressive symptoms, and fear of the virus, which were mostly related to increased preventive procedures against the virus (Bäuerle et al., 2020; French et al., 2020; Huang & Zhao, 2020; Wong et al., 2020).

Academics and faculty have largely been affected by the lockdown in terms of work time, housework routines, and childcare (Yildirim and Eslen-Ziya), and generalized anxiety disorder has been evident in this group during the COVID-19 outbreak (Huang & Zhao, 2020). Additionally, the digital pedagogies provided by their institutions have caused professional and personal disruptions (Watermeyer et al., 2020). They have further suffered from obstacles, such as the complete closure of libraries or reductions in their hours of operation (Fasae et al., 2020; Kosciejew, 2020), the inability of libraries to completely take advantage of social media to provide their services (Koulouris et al., 2020), and, finally, challenges in the access and usage of digital resources (Hendal, 2020; Mehta & Wang, 2020; Pokorna et al., 2020; Saavedra-Alamillas et al., 2020). As a result, some predict that, given the stark difference between the pre-COVID-19 environment and the post-COVID-19 world, scientific publishing (SP) and all related stakeholders will be significantly affected (Chung et al., 2020; Derrick, 2020; Kim, 2020). However, there is an expectation that unprecedented opportunities will inspire multiple researchers to investigate many facets of this crisis (da Silva et al., 2020).

Accordingly, current research hypothesizes that this health crisis had an impact on the volume and characteristics of SP in all disciplines during the first year of the COVID-19 pandemic. The focus here was on Mediterranean countries (MCs), where the author lives; these countries are marked by a wide disparity in both research production levels and infection totals, which allows for a rich comparison to realize the impact of the pandemic on scientific performance. Furthermore, this region, which includes countries located on three continents (Europe, Africa, and Asia), can represent a sample of the global situation in terms of outbreak levels and research outputs. The objectives are to (1) investigate whether there is a significant difference between the year prior to COVID-19 (2019) and the first year of the COVID-19 outbreak (2020) with regard to the annual growth rate (AGR) of SP and international collaboration (IC) as well as other characteristics, including disciplines-languages-types-rankings of journals, and (2) to explore the correlations between the cumulative totals of COVID-19 cases and SP and its characteristics.

In response to the enormous growth of scientific work associated with the novel virus, many scientometric studies have been conducted to trace the evolution of this work and its various aspects. Changes in research performance across all fields during the initial stage of the pandemic were not adequately investigated. Most analyses have focused only on the health sciences, and other disciplines were disregarded. A few studies examined the correlation between only the initial COVID-19 publications and the level of the outbreak as measured by the number of cases. Nonetheless, previous research served as an inspiration for the current investigation by outlining the key pillars that should be examined, including differences in both the volume of research and its various features, as well as their relationships with COVID-19 cases.

Some investigations highlighted the contributions of different countries and regions to COVID-19 research outputs during the first year of the pandemic. After striving to map the COVID-19 publications globally, Belli et al. (2020) found that the largest contributions were made by the United States and China. This result has been confirmed by Sahoo and Pandey (2020), Lan et al. (2020), Herrera-Viedma et al. (2020), Ho and Liu (2021), and Usman and Ho (2021). A literature review by Benjamens et al. (2020) revealed that Europe ranked first among all continents, accounting for 47.7% of the research published in four major medical journals, followed by North America, with 37.3. The types and languages of publications were also examined; research articles were the leading type, and most of the publications were published in English (da Silva et al., 2020; Al-Zaman, 2021). According to Ebadi et al. (2020), “intelligent systems”, “tools to predict”, and “diagnose COVID-19” were the most-researched areas from January to May 2020. A longer time span was analysed by Herrera-Viedma et al. (2020), who revealed an increasing volume of publications and citations related to coronavirus from 1970 to April 2020. Focusing only on nursing journals, Oh and Kim (2020) found that 60% of COVID-19 papers were published in first-quartile journals indexed in the Web of Science.

From another perspective, some researchers attempted to determine the motives for the unexpected and growing volume of COVID-19-related studies. Ho and Liu (2021) found that the most highlighted motive was the various platforms provided by the most prestigious medical journals to access the relevant outputs. Nevertheless, there are worries about the quality of this volume, which has been discussed in several studies. The analysis of papers published on PubMed and the Rxiv preprint server by Homolak et al. (2020) revealed that the drastic reduction in the time taken for editing and peer-review work during COVID-19 has necessarily affected the content quality, and there will be a need to re-review these papers later. No novel information was presented in the initial COVID-19 literature; this was inferred by Di Girolamo and Reynders (2020) after distinguishing the primary from the secondary articles indexed in PubMed. The evaluation by Pal (2021) found that the global research outburst on COVID-19 had a growth rate of 1600%. Dinis-Oliveira (2020) used the term "paperdemic” to describe this accelerated publication rate and its harmful impacts on science. To emphasize these side effects, Fernandez-Cano (2021) titled his letter to the editor "publish, publish… cursed!". In this vein, a commentary by Moradi and Abdi (2020) noted multiple corrections and erratum published regarding the significantly increased volume of COVID-19 publications. This volume also yielded several retractions, the reasons for which were mostly problems with the results, conclusions, and data (Soltani & Patini, 2020).

To the best of my knowledge (until June 2021), there is no study revealing the differences in scientific progress in all disciplines that occurred as a result of the pandemic or their correlations with the levels of the outbreak worldwide, in general, and in the Mediterranean region, in particular. The current research addresses this research gap by demonstrating how the crisis has impacted research performance in this region. This paper is the first cross-sectional comparative analysis to reveal the correlation between COVID-19 case totals and scientific progress in all disciplines (Physical Sciences, Health Sciences, Social Sciences, Life Sciences, Multidisciplinary).

## Methods

### Required data and sources

Three types of data about the MCs were collected to examine the research hypothesis.

#### The Mediterranean countries

One of the main difficulties concerning the identification of the countries in the Mediterranean region was the absence of an official government entity to identify those countries. Only the Union for the Mediterranean was found. This is an entity that unites many Mediterranean countries alongside other European countries (Union for the Mediterranean, 2020), which was not adequate for the present study. By browsing online maps of the Mediterranean region and picking the most accurate map and after seeking the advice of two experts in geopolitics,^1^ it was concluded that this region includes 23 countries bordering the Mediterranean coast (Nations Online, 2020).

#### The cumulative totals of COVID-19 cases

**Fig. 1:**
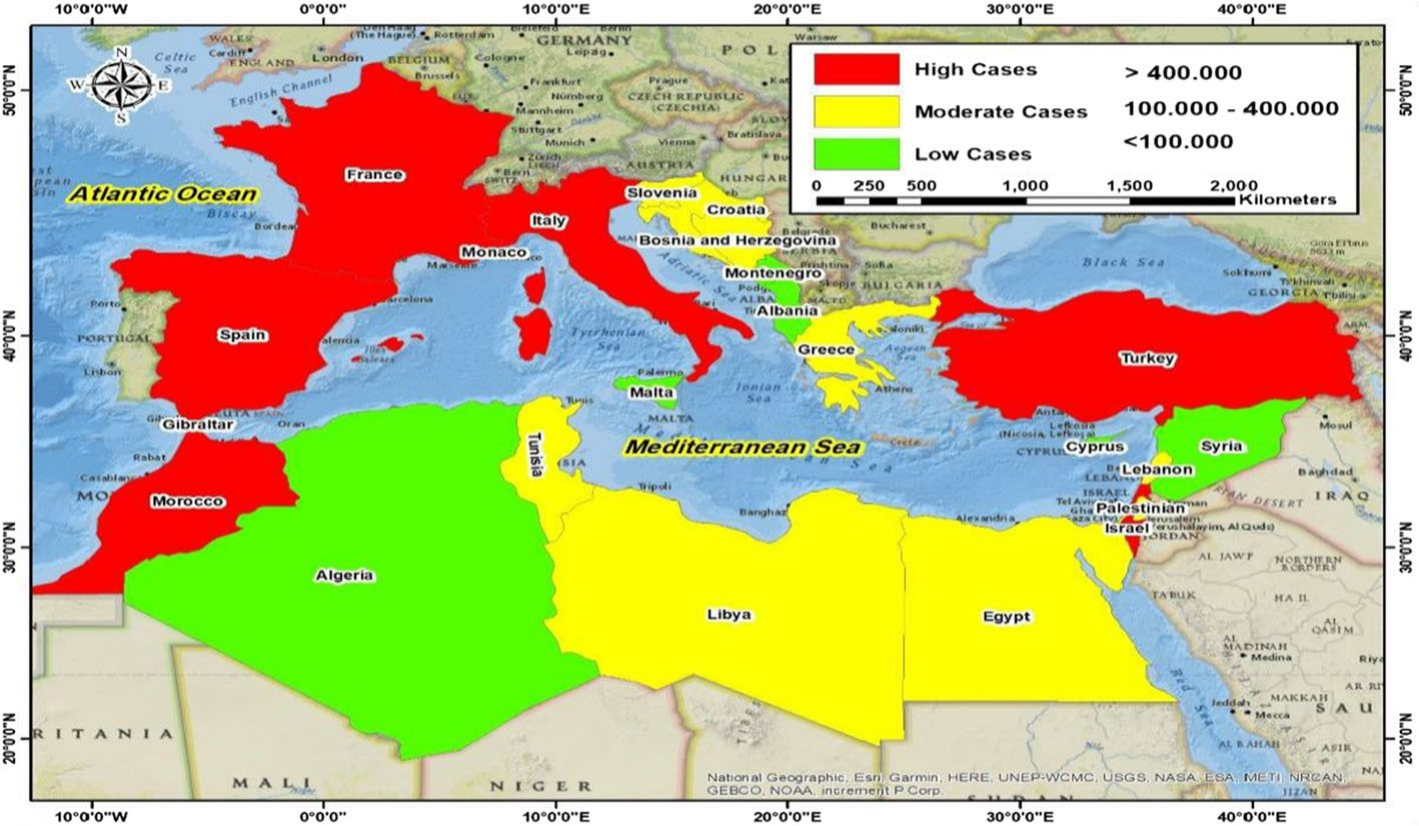
A map depicting the distribution of COVID-19 cases in Mediterranean countries from March 31 to December 31, 2020

The reported COVID-19 cases were acquired to examine their correlation with the volume and characteristics of SP. Cases were considered rather than deaths, although the actual number of COVID-19 cases may be higher than the recorded number due to factors related to the national health systems, such as the cost and extent of COVID-19 test availability (Dil et al., 2020). This is because the number of cases is the key indicator of the outbreak of the virus, as the WHO declared COVID-19 to be a PHEIC after the virus rapidly spread with a continued increasing number of confirmed cases. Furthermore, the number of cases often includes the number of deaths, which is recorded as cases before death. This is in line with initial scientometric studies on COVID-19, which have mentioned the number of cases (Di Girolamo & Reynders, 2020; Zhang et al., 2020). The WHO website was used to extract the cumulative totals of COVID-19 cases in the MCs from March 31, 2020 (the first date cases were recorded by the site), to December 31, 2020, which were announced on Jan. 1, 2021. Figure 1 demonstrates the distribution of cases in the 23 MCs. Additionally, the totals of the top 10 countries worldwide in terms of the number of cases were extracted to examine the IC between the MCs and those countries, including the United States of America, India, Brazil, Russia, France, the United Kingdom, Italy, Spain, Germany, and Colombia (World Health Organization, 2021) (see Online Resource 1, A). The number of cases represents the study's independent variable, the impact of which on the volume and characteristics of SP is measured.

#### The volume and characteristics of SP

**Fig. 2:**
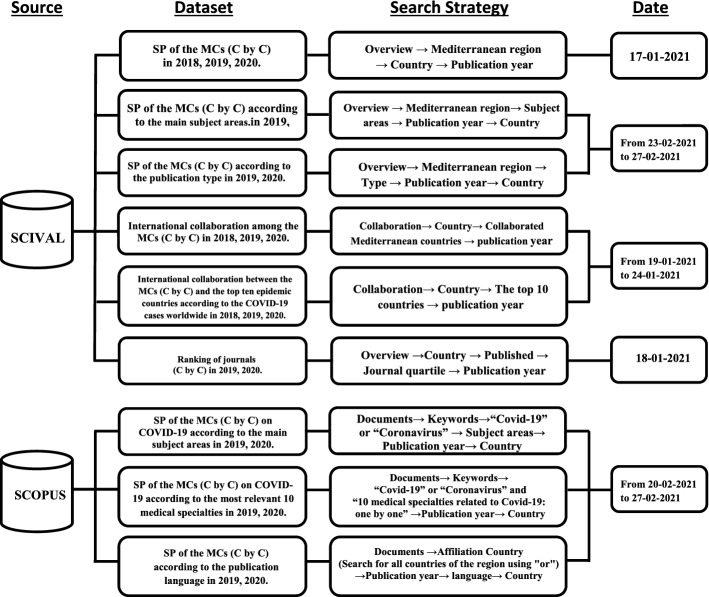
A Flowchart of the implementation of the search strategies adopted to extract the requisite data sets from SciVal and Scopus. * C by C = country by country

Both Elsevier's Scopus and Clarivate Analytics' Web of Science are major sources that bibliometricians often consult. To fulfil this paper's objectives, it was more appropriate to use Scopus because of its wider publication coverage in all disciplines and wider language coverage and because it is more up to date in indexing publications on COVID-19 (Belli et al., 2020; da Silva et al., 2020; Haghani & Bliemer, 2020; Mongeon & Paul-Hus, 2016). Furthermore, SciVal, a web-based analytics solution from Elsevier assisting those who evaluate research engagement and impact worldwide (Elsevier 2021a, 2021b), was used as an advantageous tool accessed through Scopus to obtain on-demand tailored reports for the target region. As depicted in Fig. 2, SciVal and Scopus were used for specific purposes. SciVal was first utilized to create the Mediterranean region based on a request sent by the author on Dec. 25, 2020, and it was constructed by the tool on Dec. 28, 2020. Additionally, it was used to extract the totals of both SP and IC, including the IC among MCs and the IC between the MCs and the top ten epidemic countries. To prevent data duplication, France, Italy, and Spain were excluded from the top ten list because they already belong to the MCs. In addition, SciVal helped to retrieve the region's SP characteristics by subject area, type, and journal ranking. Scopus was favoured in some search strategies because it has certain functionalities that SciVal lacks, such as the ability to use Boolean search operators to gather items related to the topic of COVID-19 in the main subject areas and medical specialties, as well as the ability to filter by language. Data for comparison between two years were needed (one-year pre-COVID-19, from January 1 to December 31, 2019, and during the first year of the COVID-19 period, from January 1 to December 31, 2020). The 2018 data were generated only to calculate the AGR of the SP volume and IC for 2019. Unquestionably, it is more efficient to capture all the data in one day to avoid database updates. However, owing to the large volume and diversity of the required data, they were extracted from January 17 to February 27, 2021, which was the shortest possible period available to the author. Moreover, given that the data collected were retrospective, it was taken into consideration that update opportunities would be rare, especially since SciVal, from which most of the data were extracted, is updated weekly every Tuesday.

There are further considerations as follows: (1) The main subject areas in both SciVal and Scopus include "Physical Sciences" (11 sub-areas), "Health Sciences" (6 sub-areas), "Social Sciences" (7 sub-areas), and "Life Sciences" (6 sub-areas). Each subject area has a sub-area called "multidisciplinary". When extracting data related to publications categorized under these main areas, a subject area filter was used. It considers "multidisciplinary" an independent subject area that includes all publications classified under "multidisciplinary" included in the four subject areas. (2) Since one document can be classified under more than one subject area, this led to higher totals for the subject area distribution than the totals of documents according to country. (3) Regarding the ten medical specialties related to COVID-19 listed by (Zhang et al., 2020), each specialty was embedded in the relevant search strategy with the main keywords of COVID-19 and coronavirus, for example, “COVID-19” or “coronavirus” and “virology”. For dual disciplines such as “Pharmacology & Pharmacy”, they were divided into two terms as follows: “COVID-19” or “coronavirus” and “Pharmacy”/“COVID-19” or “coronavirus” and “Pharmacology”.

### Statistical analysis tests

To measure the growth rates for the target years (2019, 2020), the AGR of both SP and IC in the MCs was calculated by the following equation for two periods (2018–2019 and 2019–2020) for every country. The AGR is a measurement used to express a year-on-year change in a variable as a percentage. It can be beneficial in assessing performance in any activity to monitor success or failure.

$$AGR=\frac{End\,Value-First\,Value}{First\,Value} \times 100$$

The differences between the AGRs of the two years were calculated for every country:

$$\mathrm{Difference\,of\,AGR }=2020 AGR-2019AGR$$

To study the effect of COVID-19 on the SP characteristics, the difference for each characteristic between the two years was calculated for every country.

$$\mathrm{Difference\,in\,characteristic}=\sum 2020-\sum 2019$$

Two-sample hypothesis testing was used to determine if there was a significant difference between the two years. Initially, Shapiro–Wilk and Kolmogorov–Smirnov normality tests were used to determine whether to use parametric or non-parametric tests based on the data's normality (Razali & Wah, 2011). Then, because all of the difference values were non-normal distributed, the analysis depended on the non-parametric Mann–Whitney test (Nachar, 2008) to examine the impact of COVID-19 on the calculated AGRs and SP characteristics. Additionally, correlation tests were performed to determine whether there was a positive or negative correlation between the differences calculated and the cumulative totals of COVID-19 cases in MCs. Since the data did not have a normal distribution, the non-parametric Spearman and Kendall correlations (Croux & Dehon, 2010) were used. The significance level was 10% for all tests.

### Applications and illustration design

The analysis was performed by IBM-SPSS, version 20, and all extracted test values are grouped in (Online Resource 1, B). The tables and figures were designed using Microsoft Excel 2010. All MCs are ranked in all tables in descending order based on the number of infections, and the continent symbol for each country is added in all the tables (Africa, AF; Asia, AS; Europe, EU). Regarding the meaning of the values in the charts, the positive values indicate an increase in the SP during the spread of COVID-19, while the negative values reflect its decrease. Further, the map of MCs infections was generated by ArcGIS Pro, version 10.5, Esri Inc. To distinguish the MCs, they were classified into three categories according to the recorded cases as follows: high (red), > 400,000; moderate (yellow), 100,000–400,000; and low (green), < 100,000.

## Results

This analytical section highlights the most important findings regarding the quantitative and qualitative characteristics of SP in MCs before and during the first year of the COVID-19 pandemic. It further measures the correlations between those characteristics and the number of cases recorded in the countries surveyed. The following are the results presented according to the core questions of the study.

### Are there significant differences between 2019 and 2020 in terms of the AGR of SP? Are they affected by the number of COVID-19 cases?

**Fig. 3:**
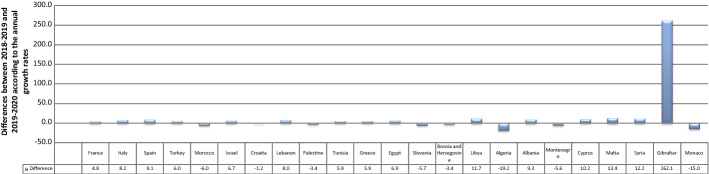
Differences between 2018–2019 and 2019–2020 according to the annual growth rates (AGRs) of scientific publishing in the Mediterranean countries. Each bar represents the change between 2019 and 2020 according to the growth rate (a percentage). The positive values indicate an increase in the annual growth rates of publications, while the negative values indicate a decrease

It is remarkable that the details provided in Table 1 regarding infection count and SP volume reveal that while France, Italy, and Spain ranked first among the MCs, Gibraltar and Monaco occupied the bottom of the list. For AGR, there was a dramatic difference, as the overall AGR of SP jumped from 3.1 to 9.4% in the first year of the COVID-19 outbreak (2020). Although the AGR dropped in eight countries, it rose in the remaining countries (Fig. 3). Algeria and Monaco had the lowest AGRs, with decreases of approximately 19% and 15%, respectively. Gibraltar had the largest AGR (262%), despite its production remaining low. This is because the AGR equation yields a percentage of an increase or decrease in publications, regardless of the volume of publications itself. Hence, the AGR achieved by this country means that its production rose by a high percentage after COVID-19, regardless of the volume of its production. On the other hand, the small AGR values in other countries (positive or negative) indicated that publications increased or declined by a low percentage. The p-value of the Mann–Whitney test was 0.106; accordingly, there was a significant difference in the AGR between 2019 and 2020. The medians of the two years were 8.73% and 11.80%, respectively. Hence, the AGR of the SP increased during the spread of COVID-19. Contrary to expectations, the number of COVID-19 cases did not have a significant impact on these differences. This is because the p-value of the Spearman and Kendall correlations was greater than 0.1. In other words, an increase or decrease in the number of infections had no significant positive or negative impact on SP during the pandemic.

**Table 1:** The annual growth rate of scientific publishing in the Mediterranean countries before and during COVID-19

Regional rank	*Global rank	Continent /country	No. of cases	N0. of publication			AGR 2019	AGR2020
				**2018	2019	2020		
1	5	EU/France	2,576,420	124,196	121,049	123,754	− 2.5	2.2
2	7	EU/Italy	2,107,166	123,465	127,876	142,969	3.6	11.8
3	8	EU/Spain	1,893,502	98,589	101,396	113,471	2.8	11.9
4	13	EU/Turkey	1,394,314	45,593	49,607	56,955	8.8	14.8
5	31	AF/Morocco	439,193	7,545	8,661	9,425	14.8	8.8
6	34	AS/Israel	408,277	22,907	23,203	25,067	1.3	8.0
7	46	EU/Croatia	212,007	7,688	7,826	7,869	1.8	0.5
8	52	AS/Lebanon	181,503	3,591	3,936	4,630	9.6	17.6
9	58	AS/Palestine	155,365	870	1,025	1,173	17.8	14.4
10	63	AF/Tunisia	139,140	8,598	8,240	8,384	− 4.2	1.7
11	64	EU/Greece	138,850	20,405	20,794	22,425	1.9	7.8
12	65	AF/Egypt	138,062	22,029	25,725	31,813	16.8	23.7
13	70	EU/Slovenia	123,950	6,506	6,941	7,006	6.7	0.9
14	74	EU/Bosnia and Herzegovina	110,985	1,441	1,570	1,657	9.0	5.5
15	76	AF/Libya	100,277	503	554	675	10.1	21.8
16	77	AF/Algeria	99,610	7,768	8,631	7,937	11.1	− 8.0
17	89	EU/Albania	58,316	530	549	620	3.6	12.9
18	94	EU/Montenegro	48,231	511	576	617	12.7	7.1
19	106	AS/Cyprus	22,651	3,023	3,287	3,908	8.7	18.9
20	124	EU/Malta	12,774	992	974	1,087	− 1.8	11.6
21	127	AS/Syria	11,434	502	576	731	14.7	26.9
22	170	EU/Gibraltar	1,973	11	6	19	− 45.5	216.7
23	179	EU/Monaco	875	180	240	284	33.3	18.3
Total			10,374,875	507,443	523,242	572,476	3.1	9.4

*(Global rank) refers to the ranking of countries in terms of COVID-19 cases globally

**The 2018 values were extracted only to calculate the AGR for 2019, but they are out of the study scope

### Are there significant differences between 2019 and 2020 in terms of the AGR of IC among MCs and between MCs and the top 10 countries in terms of COVID-19 infections? Are they affected by the number of COVID-19 cases?

**Fig. 4:**
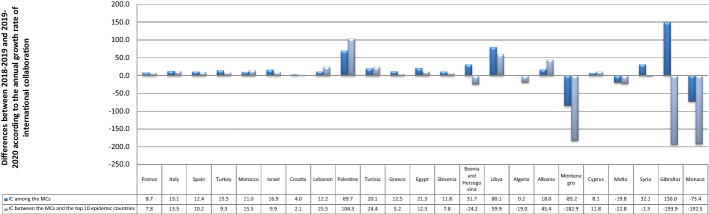
Differences between 2018–2019 and 2019–2020 according to the annual growth rate of international collaboration (1) among the Mediterranean countries and (2) between the Mediterranean countries and the top 10 countries affected by the pandemic. Each bar represents the change between 2019 and 2020 according to the growth rate (a percentage) of collaboration. The positive values indicate an increase in the annual growth rates of international collaboration, while the negative values indicate a decrease

Table 2 shows that the overall AGR of IC increased dramatically between 2019 and 2020, both among MCs (from 3.1 to 15.1%) and between MCs and the top ten epidemic countries (from 2.7 to 12.8%). As with the total SP, France, Italy, and Spain outpaced all of the MCs in both forms of IC. The USA, the United Kingdom, and Germany were the top collaborating countries, especially with Italy and France (for more details see Online Resource 1, C and 1, D). For the IC among MCs, Montenegro, Monaco, and Malta witnessed a decrease during the pandemic in the AGR of IC by approximately 85%, 73.4%, and 19.8%, respectively. However, the AGR improved in the rest of the countries and remained constant in Algeria. The highest rates were reported in Gibraltar, Libya, and Palestine, rising by 150%, 80.1%, and 69.7%, respectively. In terms of IC with the top 10 countries, the AGR dropped in seven countries, including Gibraltar (193.9%), Monaco (192.5%), Montenegro (182.9%), Bosnia and Herzegovina (24.2%), Malta (22.8%), Algeria (19%), and Syria (2.3%). Nevertheless, it increased in other MCs. Palestine, Libya, and Albania had the highest AGRs, jumping by 104.3%, 59.9%, and 45.4%, respectively, as shown in Fig. 4. The result of the Mann–Whitney test was zero for the IC among MCs; there was a significant difference between the two years. The medians of both years were 3.76% and 16.12%, proving that it progressed during 2020. Similarly, IC between MCs and the top 10 countries increased. The p-value of the test was 0.097, and there was a significant difference between the median values of the two years, 4.03% and 14.36%. The correlation tests also showed that the number of infections had no impact on the differences in IC among MCs (p-value > 0.1). They did, however, confirm that IC between the MCs and the top ten epidemic countries was influenced by the number of infections (p-value < 0.1). The relationship appeared moderately positive, implying that an increase in infections enhanced the IC between MCs and the top ten countries.

**Table 2:** The annual growth rate of international collaboration in the Mediterranean countries before and during COVID-19

Regional rank	Continent /country	No. of Cases	IC among the MCs					IC between the MCs and the top 10 epidemic countries				
			No. of publication			AGR		No. of publication			AGR	
			2018	2019	2020	2019	2020	2018	2019	2020	2019	2020
1	EU/France	2,576,420	30,506	30,857	33,889	1.2	9.8	50,621	50,637	54,597	0.0	7.8
2	EU/Italy	2,107,166	28,100	29,182	34,114	3.9	16.9	46,926	48,116	55,839	2.5	16.1
3	EU/Spain	1,893,502	23,243	24,118	28,007	3.8	16.1	37,175	38,335	43,450	3.1	13.3
4	EU/Turkey	1,394,314	6,728	7,103	8,597	5.6	21.0	8,923	9,614	11,251	7.7	17.0
5	AF/Morocco	439,193	3,051	3,149	3,595	3.2	14.2	1,543	1,525	1,744	− 1.2	14.4
6	AS/Israel	408,277	5,711	5,628	6,500	− 1.5	15.5	11,374	11,590	12,960	1.9	11.8
7	EU/Croatia	212,007	4,206	4,315	4,601	2.6	6.6	3,490	3,548	3,682	1.7	3.8
8	AS/Lebanon	181,503	1,724	1,916	2,363	11.1	23.3	1,245	1,362	1,837	9.4	34.9
9	AS/Palestine	155,365	700	535	782	− 23.6	46.2	511	340	581	− 33.5	70.9
10	AF/Tunisia	139,140	3,662	3,470	3,984	− 5.2	14.8	919	956	1,228	4.0	28.5
11	EU/Greece	138,850	9,176	9,408	10,826	2.5	15.1	10,356	10,538	11,276	1.8	7.0
12	AF/Egypt	138,062	3,278	3,596	4,711	9.7	31.0	5,812	6,492	8,051	11.7	24.0
13	EU/Slovenia	123,950	3,825	3,825	4,278	0.0	11.8	3,027	3,029	3,267	0.1	7.9
14	EU/Bosnia and Herzegovina	110,985	756	680	827	− 10.1	21.6	278	347	349	24.8	0.6
15	AF/Libya	100,277	288	255	430	− 11.5	68.6	207	192	293	− 7.2	52.6
16	AF/Algeria	99,610	3,209	3,365	3,534	4.9	5.0	802	1,026	1,118	27.9	9.0
17	EU/Albania	58,316	384	445	596	15.9	33.9	143	159	249	11.2	56.6
18	EU/Montenegro	48,231	281	681	1,070	142.3	57.1	91	349	700	283.5	100.6
19	AS/Cyprus	22,651	2,798	2,974	3,401	6.3	14.4	2,268	2,235	2,467	− 1.5	10.4
20	EU/Malta	12,774	719	853	843	18.6	− 1.2	498	592	569	18.9	− 3.9
21	AS/Syria	11,434	142	167	250	17.6	49.7	128	157	189	22.7	20.4
22	EU/Gibraltar	1,973	4	4	10	0.0	150.0	3	11	19	266.7	72.7
23	EU/Monaco	875	165	294	308	78.2	4.8	107	296	249	176.6	− 15.9
Total		10,374,875	132,656	136,820	157,516	3.1	15.1	186,447	191,446	215,965	2.7	12.8

### Are there significant differences between 2019 and 2020 in terms of the characteristics of SP? Are they affected by the number of COVID-19 cases?

#### Main subject areas

**Fig. 5:**
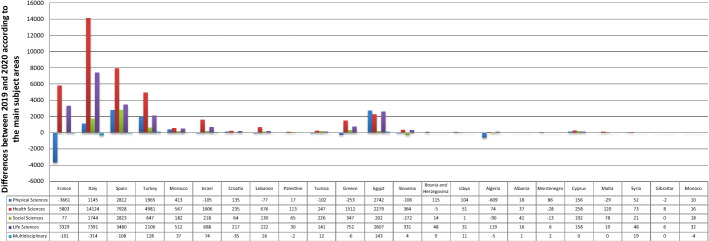
Differences between 2019 and 2020 according to the main subject areas of scientific publishing in the Mediterranean countries. Each bar represents the change between 2019 and 2020 according to the number of publications in the subject area. The positive values indicate an increase in the volume, while the negative values indicate a decrease

As Table 3 and Fig. 5 indicate, only the life sciences witnessed a rise in publications in all 23 MCs during the first year of the COVID-19 period. The largest differences were achieved by Italy, Spain, France, Egypt, and Turkey, with 7391, 3480, 3329, 2607, and 2106 publications, respectively. The other subject areas experienced an increase in most of the MCs. For the physical sciences, the largest differences were in Spain, Egypt, Turkey, and Italy, where SP increased by 2812, 2742, 1965, and 1145 publications, respectively. Additionally, Italy, Spain, France, Turkey, and Egypt had the largest increases in the health sciences, with increases of 14,124, 7928, 5803, 4981, and 2279, respectively. Spain and Italy stood out in the social sciences, with increases of 2823 and 1744, respectively. Egypt (143) and Turkey (128) ranked first and second in the multidisciplinary sciences. The p-value of the Mann–Whitney test of all the subject areas was greater than 0.1, i.e., COVID-19 did not significantly affect SP in those subject areas. For the physical and multidisciplinary sciences, the p-value of the correlation tests was greater than 0.1, i.e., the number of cases did not significantly affect the differences in SP in the MCs in these two areas. The health, social, and life sciences were significantly affected (p-value ˂ 0.1). According to the correlation coefficient values, there was a strong positive relation; the number of publications in these three sciences grew as the number of infections increased.

**Table 3:** Scientific publishing before and during COVID-19 in Mediterranean countries according to the main subject areas

Regional rank	Continent /country	No. of cases	Physical Sciences		Health Sciences		Social Sciences		Life Sciences		Multidisciplinary	
			2019	2020	2019	2020	2019	2020	2019	2020	2019	2020
1	EU/France	2,576,420	66,962	63,301	33,343	39,146	16,932	17,009	28,075	31,404	3,209	3,108
2	EU/Italy	2,107,166	64,682	65,827	41,498	55,622	20,035	21,779	31,648	39,039	2,793	2,479
3	EU/Spain	1,893,502	48,568	51,380	29,452	37,380	21,460	24,283	24,886	28,366	2,668	2,560
4	EU/Turkey	1,394,314	24,703	26,668	17,240	22,221	7,301	7,948	9,349	11,455	468	596
5	AF/Morocco	439,193	6,819	7,232	1,064	1,631	1,123	1,305	1,053	1,565	100	137
6	AS/Israel	408,277	10,435	10,330	6,865	8,471	5,309	5,527	5,366	6,054	637	711
7	EU/Croatia	212,007	3,591	3,726	2,203	2,438	1,955	2,019	1,636	1,853	137	102
8	AS/Lebanon	181,503	1,815	1,738	1,524	2,200	683	813	800	1,022	70	86
9	AS/Palestine	155,365	628	645	207	320	215	280	167	197	32	30
10	AF/Tunisia	139,140	5,734	5,632	1,498	1,745	999	1,225	1,546	1,687	83	95
11	EU/Greece	138,850	11,374	11,121	6,741	8,253	3,410	3,757	4,656	5,408	272	266
12	AF/Egypt	138,062	15,242	17,984	6,809	9,088	1,772	1,974	7,125	9,732	522	665
13	EU/Slovenia	123,950	3,906	3,800	1,380	1,744	1,725	1,453	1,357	1,688	153	157
14	EU/Bosnia and Herzegovina	110,985	832	947	506	501	324	338	220	268	10	19
15	AF/Libya	100,277	362	466	103	154	80	81	115	146	9	20
16	AF/Algeria	99,610	7,554	6,945	498	572	731	641	1,121	1,240	104	99
17	EU/Albania	58,316	289	307	171	208	114	155	127	143	7	8
18	EU/Montenegro	48,231	303	389	151	123	154	141	147	153	4	6
19	AS/Cyprus	22,651	1,696	1,852	883	1,141	927	1,119	595	753	47	47
20	EU/Malta	12,774	472	443	318	438	218	296	170	189	17	17
21	AS/Syria	11,434	295	347	201	274	40	61	186	234	9	28
22	EU/Gibraltar	1,973	5	3	0	8	2	2	3	9	1	1
23	EU/Monaco	875	97	107	88	104	30	48	70	102	14	10
Total		10,374,875	272,773	281,190	152,743	193,782	85,539	92,254	120,418	142,707	11,366	11,247

#### The COVID-19 topic in the main subject areas

**Fig. 6:**
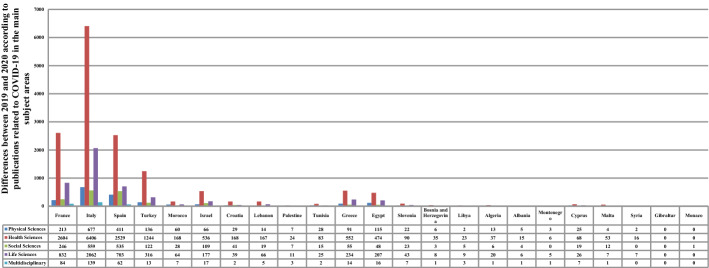
Differences between 2019 and 2020 according to the Mediterranean publications related to COVID-19 in the main subject areas. Each bar represents the change between 2019 and 2020 according to the number of COVID-19 publications in the subject area

Concerning the SP related to COVID-19, the physical, health, and life sciences remained unchanged during 2020 in two countries—Gibraltar and Monaco—although it grew in the rest of the countries. Italy, Spain, and France had the greatest positive variations, increasing by 677 in the physical sciences, 6406 in the health sciences, and 2062 in the life sciences; 411 in the physical sciences, 2529 in health sciences, and 703 in life sciences; and 213 in the physical sciences, 2604 in the health sciences, and 832 in the life sciences, respectively. The three leading countries also led in both the social sciences (Italy, 559; Spain, 535; and France, 246) and multidisciplinary sciences (Italy, 139; France, 84; and Spain, 62), whereas a few countries witnessed no change in the two subject areas, as shown in Table 4 and Fig. 6. The tests proved that the pandemic had a substantial impact on the SP tagged under COVID-19 (p-value < 0.1). As predicted, for all of the sciences, the infection number had a significant effect on the SP related to COVID-19 (p-value < 1). The correlation coefficients indicated a strong positive relationship; that is, the publications tagged under COVID-19 increased as the number of infections increased.

**Table 4:** Scientific publishing before and during COVID-19 in Mediterranean countries according to publications related to COVID-19 in the main subject areas

Regional rank	Country	No. of cases	Physical Sciences		Health Sciences		Social Sciences		Life Sciences		Multidisciplinary	
			2019	2020	2019	2020	2019	2020	2019	2020	2019	2020
1	EU/France	2,576,420	3	216	14	2618	0	246	23	855	1	85
2	EU/Italy	2,107,166	2	679	21	6427	1	560	15	2077	0	139
3	EU/Spain	1,893,502	1	412	14	2543	0	535	11	714	0	62
4	EU/Turkey	1,394,314	1	137	8	1252	0	122	6	322	0	13
5	AF/Morocco	439,193	0	60	2	170	0	28	2	66	0	7
6	AS/Israel	408,277	0	66	0	536	0	109	0	177	0	17
7	EU/Croatia	212,007	0	29	1	169	0	41	1	40	0	2
8	AS/Lebanon	181,503	0	14	6	173	0	19	6	72	0	5
9	AS/Palestine	155,365	0	7	0	24	0	7	0	11	0	3
10	AF/Tunisia	139,140	0	28	2	85	0	15	3	28	0	2
11	EU/Greece	138,850	0	91	0	552	0	55	3	237	0	14
12	AF/Egypt	138,062	1	116	20	494	0	48	21	228	2	18
13	EU/Slovenia	123,950	0	22	0	90	0	23	0	43	0	7
14	EU/Bosnia and Herzegovina	110,985	0	6	0	35	0	3	0	8	0	1
15	AF/Libya	100,277	0	2	0	23	0	5	0	9	0	3
16	AF/Algeria	99,610	1	14	1	38	0	6	1	21	0	1
17	EU/Albania	58,316	0	5	0	15	0	4	0	6	0	1
18	EU/Montenegro	48,231	0	3	0	6	0	0	0	5	0	1
19	AS/Cyprus	22,651	0	25	0	68	0	19	0	26	0	7
20	EU/Malta	12,774	0	4	1	54	0	12	0	7	0	1
21	AS/Syria	11,434	0	2	0	16	0	0	0	7	0	0
22	EU/Gibraltar	1973	0	0	0	0	0	0	0	0	0	0
23	EU/Monaco	875	0	0	0	0	0	1	0	0	0	0
Total		10,374,875	9	1938	90	15,388	1	1858	92	4959	3	389

#### The COVID-19 topic in the most relevant medical specialties

**Fig. 7:**
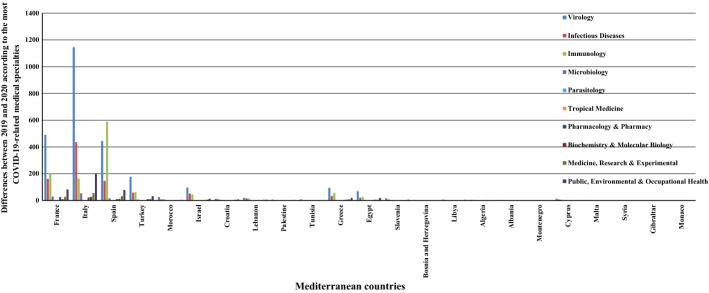
Differences between 2019 and 2020 according to the Mediterranean publications classified under the most COVID-19-related medical specialties. Each bar represents the change between 2019 and 2020 according to the number of publications in the COVID-19-related medical specialty

Overall, the SP in most COVID-19-related medical specialties increased. A rise in parasitology and tropical medicine publications was absent or very rare (1 or 2 publications in 4 countries), but it was concentrated, for other specialties, in seven countries: Italy, Spain, France, Turkey, Israel, Greece, and Egypt. The largest increases in virology, infectious diseases and public, environmental and occupational health were recorded by Italy (1145, 435, and 201), while the most marked increase (588) in immunology came from Spain (see Table 7 in the Appendix and Fig. 7). Except for parasitology and tropical medicine, all the specialties analysed were affected by the emergence of the virus (p-value < 0.1). While it was thought that the number of infections would have a significant impact on all 10 core medical specialties associated with COVID-19, the tests revealed the opposite. For parasitology and tropical medicine, the number of cases was weakly correlated with SP in both disciplines (p-value > 0.1). The p-value for the others was less than 0.1, and the values of the correlation coefficients proved that these specialties were positively correlated with the case count.

#### Languages

**Fig. 8:**
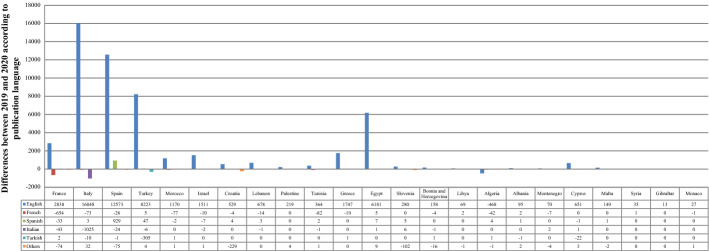
Differences between 2019 and 2020 according to the languages of scientific publishing in the Mediterranean countries. Each bar represents the change between 2019 and 2020 according to the number of publications in the language. The positive values indicate an increase in the volume, while the negative values indicate a decrease

English was, as predicted, the most dominant language for publishing. English publications saw a boost after COVID-19 in all MCs except Algeria, where they dropped by 468. With the exception of Spanish publications, which increased by 929 in Spain, publications in other languages decreased in some countries and stabilized in others during 2020. Italy led publishing in English with a difference of 16,048 papers, followed by Spain (12,573), Turkey (8223), Egypt (6181), and France (2838), as depicted in Table 5 and Fig. 8. Nonetheless, the publication languages were not affected by the emergence of COVID-19 (p-value˃0.1). The p-value of the correlation tests was greater than 0.1 for Spanish, Turkish, and other languages, indicating that the number of cases did not significantly affect the SP in these languages. However, this number had a considerable influence on SP in English, French, and Italian (p-value < 0.1). According to the values of the correlation coefficients, there was a strong positive relationship for English publications, a moderate negative relationship for French publications, and a strong negative relationship for Italian publications. Consequently, the more cases of infection there were, the more publications produced in English and the fewer in French and Italian there were.

**Table 5:** Scientific publishing before and during COVID-19 in Mediterranean countries according to the publication language

Regional rank	Continent /country	No. of cases	English		French		Spanish		Italian		Turkish		Others	
			2019	2020	2019	2020	2019	2020	2019	2020	2019	2020	2019	2020
1	EU/France	2,576,420	114,723	117,561	10,412	9758	310	277	92	49	3	5	337	263
2	EU/Italy	2,107,166	123,872	139,920	251	178	378	381	3873	2848	11	1	218	250
3	EU/Spain	1,893,502	91,751	104,324	229	203	13,543	14,472	67	43	1	0	405	330
4	EU/Turkey	1,394,314	47,517	55,740	65	70	60	107	11	5	2418	2113	42	46
5	AF/Morocco	439,193	8337	9507	465	388	8	6	0	0	0	1	2	3
6	AS/Israel	408,277	23,244	24,755	32	22	14	7	3	1	0	0	48	49
7	EU/Croatia	212,007	6798	7327	8	4	3	7	10	10	0	0	1089	860
8	AS/Lebanon	181,503	3914	4592	72	58	1	4	1	0	0	0	2	2
9	AS/Palestine	155,365	1022	1241	1	1	1	1	0	0	0	0	2	6
10	AF/Tunisia	139,140	8026	8390	401	319	4	6	2	1	0	0	1	2
11	EU/Greece	138,850	20,640	22,387	33	23	16	16	4	4	0	1	178	178
12	AF/Egypt	138,062	25,741	31,922	26	31	27	34	0	1	0	0	19	28
13	EU/Slovenia	123,950	6442	6722	9	9	0	5	5	11	0	0	541	439
14	EU/Bosnia and Herzegovina	110,985	1539	1697	4	0	0	0	1	0	1	2	53	37
15	AF/Libya	100,277	154	223	0	2	0	0	0	0	0	0	1	0
16	AF/Algeria	99,610	8515	8047	185	143	3	7	1	1	0	1	6	5
17	EU/Albania	58,316	548	643	1	3	2	3	1	1	1	0	1	3
18	EU/Montenegro	48,231	559	629	7	0	1	1	1	3	0	0	23	19
19	AS/Cyprus	22,651	3262	3913	5	5	3	2	0	1	33	11	23	26
20	EU/Malta	12,774	985	1134	0	0	1	2	0	0	0	0	3	1
21	AS/Syria	11,434	92	127	0	1	0	0	0	0	0	0	0	0
22	EU/Gibraltar	1973	6	19	0	0	0	0	0	0	0	0	0	0
23	EU/Monaco	875	241	268	7	6	0	0	0	0	0	0	0	1
Total		10,374,875	497,928	551,088	12,213	11,224	14,375	15,338	4072	2979	2468	2135	2994	2548

#### Types

**Fig. 9:**
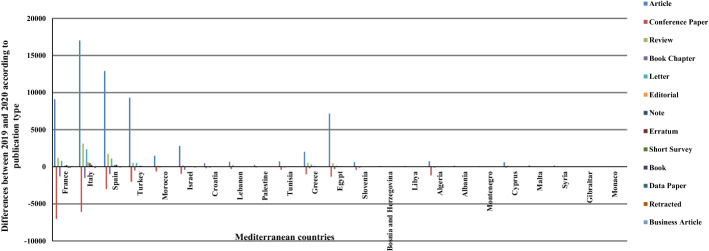
Differences between 2019 and 2020 according to the types of scientific publishing in the Mediterranean countries. Each bar represents the change between 2019 and 2020 according to the number of publications in the type. The positive values indicate an increase in the volume, while the negative values indicate a decrease

Likewise, the spread of COVID-19 had no significant effect on the publication type (p-value > 0.1). Table 8 in the Appendix and Fig. 9 reveal that conference papers decreased in all the MCs except for Bosnia and Herzegovina, Libya, and Montenegro, which saw increases. Additionally, book chapters declined in all the MCs but Montenegro and Syria. Retractions and business articles presented only minor variations or remained constant in almost all the countries. On the other hand, articles, letters, and reviews increased in most countries. Publication type was partially influenced by the case count. The p-value of the tests was more than 0.1 for retractions and business articles, reflecting that publications of either type were not significantly affected by the number of cases. However, the case count had an impact on other types (p-value < 0.1). The correlation coefficients for articles, review papers, letters, editorials, notes, errata, and data papers showed a strong or moderately positive relationship. However, there was a strong or moderately negative relationship for conference papers, book chapters, short surveys, and books.

#### Journal rankings

**Fig. 10:**
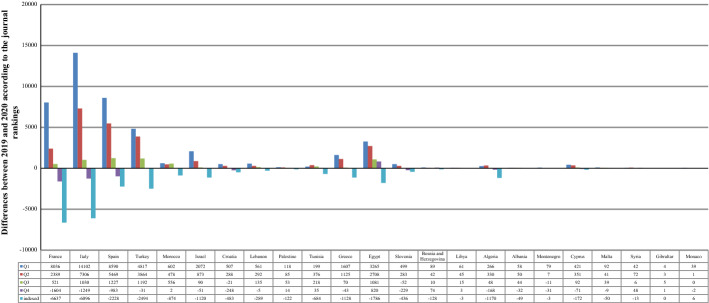
Differences between 2019 and 2020 according to the rankings of the journals in which the Mediterranean publications were published. Each bar represents the change between 2019 and 2020 according to the number of publications in the quartile. The positive values indicate an increase in the volume, while the negative values indicate a decrease

Considering the rankings of the journals as a measure of publication quality, it is quite impressive that Q1 and Q2 journals witnessed a rise in all the countries after COVID-19. The greatest increases were in Italy (14,102 in Q1, 7306 in Q2), Spain (8590 in Q1, 5469 in Q2), France (8036 in Q1, 2389 in Q2), Turkey (4817 in Q1, 3864 in Q2), and Egypt (3265 in Q1, 2708 in Q2). Other rankings, on the other hand, saw a drop in several countries. For the Q3 journals, the greatest differences were in Spain, Turkey, Egypt, and Italy, where the publications increased by 1227, 1192, 1081, and 1030, respectively. The SP in Q4 and indexed journals decreased sharply in most of the MCs. Conversely, the greatest difference in Q4 journals, 820, was seen in Egypt, whereas Monaco's publications in indexed journals increased by only 6 (Table 6 and Fig. 10). Nevertheless, the tests proved that COVID-19 did not significantly affect SP in any of the ranks of international journals (p-value > 0.1). The correlation tests revealed that the relationship was strong or moderately positive for Q1, Q2, and Q3 journals, whereas it was strong or moderately negative for Q4 and indexed journals. As such, increases in the case count resulted in more publications in Q1, Q2, and Q3 journals and fewer publications in Q4 and indexed journals (p-value < 0.1).

**Table 6:** Scientific publishing before and during COVID-19 in Mediterranean countries according to the journal rankings

Regional rank	Continent /country	No. of cases	Q1		Q2		Q3		Q4		Indexed in Scopus	
			2019	2020	2019	2020	2019	2020	2019	2020	2019	2020
1	EU/France	2,576,420	61,838	69,874	21,545	23,934	11,974	12,495	11,589	9985	14,103	7466
2	EU/Italy	2,107,166	59,184	73,286	29,195	36,501	15,963	16,993	9479	8230	14,055	7959
3	EU/Spain	1,893,502	51,915	60,505	19,836	25,305	13,427	14,654	9149	8166	7069	4841
4	EU/Turkey	1,394,314	14,103	18,920	12,867	16,731	10,871	12,063	6551	6520	5215	2721
5	AF/Morocco	439,193	1701	2303	1841	2319	2023	2579	1461	1463	1635	761
6	AS/Israel	408,277	13,045	15,117	4928	5801	1867	1957	830	779	2533	1413
7	EU/Croatia	212,007	2590	3097	1648	1936	1437	1416	1266	1018	885	402
8	AS/Lebanon	181,503	1529	2090	957	1249	583	718	245	240	622	333
9	AS/Palestine	155,365	328	446	292	377	146	199	98	112	161	39
10	AF/Tunisia	139,140	2456	2655	2325	2701	1540	1758	586	621	1333	649
11	EU/Greece	138,850	8977	10,584	4888	6013	2763	2833	1437	1394	2729	1601
12	AF/Egypt	138,062	8505	11,770	6650	9358	4951	6032	2675	3495	2944	1158
13	EU/Slovenia	123,950	3000	3499	1552	1835	1064	1012	669	440	656	220
14	EU/Bosnia and Herzegovina	110,985	294	383	339	381	325	335	372	446	240	112
15	AF/Libya	100,277	153	214	122	167	105	120	59	62	115	112
16	AF/Algeria	99,610	1781	2047	2137	2467	1896	1944	1140	972	1677	507
17	EU/Albania	58,316	122	180	108	158	116	160	123	91	80	31
18	EU/Montenegro	48,231	158	237	137	144	114	103	120	89	47	44
19	AS/Cyprus	22,651	1395	1816	688	1039	461	553	300	229	443	271
20	EU/Malta	12,774	342	434	268	309	122	161	91	82	151	101
21	AS/Syria	11,434	126	168	150	222	157	163	109	157	34	21
22	EU/Gibraltar	1973	5	9	1	4	0	5	0	1	0	0
23	EU/Monaco	875	162	201	41	42	21	21	9	7	7	13
Total		10,374,875	233,709	279,835	112,515	138,993	71,926	78,274	48,358	44,599	56,734	30,775

In summary, neither the number of publications nor the growth rates showed an upwards trend between 2019 and 2020 in some countries. Statistical tests also showed that some increases and decreases were not significant. According to the findings, the first year of COVID-19 witnessed significant differences in terms of the volume of SP, the two kinds of IC, the SP tagged under COVID-19, and most of the related medical specialties. There was also a positive relationship between the number of COVID-19 cases and the following: the IC between the MCs and the top ten epidemic countries, the SP in the health, social, and life sciences, SP classified under COVID-19 in all fields, most of the relevant medical specialties, publications in the English language, most types of publications (articles, review papers, letters, editorials, notes, errata, and data papers), and publishing in Q1, Q2, and Q3 journals. The case count was negatively correlated with publications in the French and Italian languages, some publication types (conference papers, book chapters, short surveys, and books), and publishing in Q4 and indexed journals.

## Discussion

This study provided a scientometric interpretation of the impact of the COVID-19 crisis on SP in the Mediterranean region. It answered an essential question as to whether this pandemic has inspired academics to conduct more research to address the pandemic's dimensions and potential consequences, or, on the contrary, has led them to fear and anxiety and reduced their productivity as they have confronted the outbreak socially, psychologically and even economically.

One of the methods used to identify the implications of COVID-19 was to calculate the AGRs of 2019 and 2020 for SP and IC and obtain the differences between the two years. Pal (2021) and Sahoo and Pandey (2020) also applied this method to measure the pandemic's impacts. Both found tremendous growth in COVID-19 research since January 2020. This study is consistent with their findings by revealing significant growth in SP, from 3.1% to 9.4% in the MCs during the first year of COVID-19 across all disciplines, although the AGR declined in eight countries. The AGR of IC also significantly increased in most MCs, by 12% for IC among MCs and 10% for IC with the top ten epidemic countries. Although they did not achieve the highest growth rates during 2020, Italy, France, and Spain, respectively, occupied the top of the SP and IC lists and the other lists of the SP characteristics. This is consistent with the findings of the latest papers examining the features of the global COVID-19 literature, which revealed the advanced positions that these countries occupy, especially Italy, which has often been ranked third worldwide after the U.S.A. and China (da Silva et al., 2020; Ho & Liu, 2021; Pal, 2021; Usman & Ho, 2021). The following four positions were occupied in various orders by Turkey, Egypt, Israel, and Greece. Interestingly, countries that are characterized by low production and placed at the bottom of the SP list (e.g., Malta, Gibraltar, and Monaco) recorded either the highest or lowest growth rates. This suggests that these countries were the most influenced by the pandemic, either positively or negatively, i.e., the pandemic was a motivator for some of these countries and a hurdle for others. The differentials between countries in productivity, especially during the COVID-19 era, have been explained in many studies. The research contribution from nations with large economies has been higher than that from nations with small or medium-sized economies (Mukherjee, 2020). Sociologically, it is expected that COVID-19 will increase health, education, and labour disparities among citizens all over the world (Rodríguez-Bailón, 2020). Consequently, these differences may also appear in SP.

In terms of subjects, SP generally witnessed a rise at the three levels, for which differences were examined. The most distinct differences were achieved by Italy in the subject area of health sciences (14,124), health sciences tagged as COVID-19-related (6406), and virology (1145). This indicates that the serious epidemiological situation that Italy confronted during 2020 was a huge trigger for generating more publications in health sciences. However, these differences were not statistically significant for the main subject areas, whereas they were positively influential for COVID-19-tagged literature and the majority of the COVID-19-related medical specialties. The number of COVID-19 publications increased dramatically in the health sciences, followed by the life, physical, social, and interdisciplinary sciences. This finding is somewhat in line with Fernandez-Cano (2021)’s list of core subject areas, which include Science and Technology, Social Sciences, and Art and Humanities. Moreover, the most significant differences in most COVID-19-relevant medical disciplines were found in virology, immunology, and infectious diseases, which vary slightly from the rankings of Zhang et al. (2020)—virology and infectious diseases.

The reasons for the 2020 growth have been interpreted by some analyses of the COVID-19 research as follows: the virus's spread and the losses it has caused, which have prompted researchers to race to discover its nature and address novel related topics; a shift in publishing practices, the most significant of which is the reduction in the time required for editing and review work; the encouragement of governments and funding agencies; and finally, more available time for scientists to conduct research as a result of their home isolation and the greater reliance on new forms of scientific communication, such as preprint repositories and posting of preliminary results via social media (Chung et al., 2020; da Silva, 2020; Helliwell et al., 2020; Ho & Liu, 2021; Homolak et al., 2020; Koerber, 2020; Kun, 2020; Sahoo & Pandey, 2020).

Although COVID-19 had no effect on publication languages, the study revealed the English language's dominance in publications from the MCs. Following English, the most widely used languages were Spanish, French, and Italian, which is consistent with Al-Zaman (2021) and da Silva et al. (2020) who found that English ranked first worldwide and Spanish ranked third during the initial period of the pandemic. The statistical tests also showed that the virus had no effect on publication types in 2020. Nonetheless, there are some positive and negative differences between the two studied years that should be discussed in light of the consequences of the pandemic that affect the scientific community. In agreement with Al-Zaman (2021), Sahoo and Pandey (2020), and da Silva et al. (2020), the massive rise of articles represented this type's unrivalled dominance. This is definitely due to articles’ wider acceptance as the principal means of disseminating knowledge, which enables more scientists to share information about this crisis (Oh & Kim, 2020). Further, the emergence of the COVID-19 topic prompted a boost in letters to editors. This is because they are easier and faster to write and edit, represent a platform for reviewing the latest results, and highlight future research prospects (Turki et al., 2018). The drop in conferences was predicted as a result of the travel ban and prohibition of events imposed by most countries during 2020 to prevent the spread of the virus. The decline in retractions, on the other hand, was surprising in light of the phenomenon of the acceleration of publishing reported by da Silva (2020); Soltani and Patini (2020), and Moradi and Abdi (2020), a potential consequence of which was an increase in both retractions and errata. This phenomenon itself may also explain the increase in publications in higher-ranked journals in 2020, except in the Q4 and indexed journals in Scopes. This is despite the evidence that the pandemic had no effect on SP according to these ranks. Largely in line with Oh and Kim (2020) who studied COVID-19 publications in nursing journals, the MCs' documents were published in Q1, Q2, Q3, Q4, and indexed journals, respectively. The first- and second-quartile journals accounted for 73% of the 2020 publications, implying the quality that characterized the literature from MCs during the first year of COVID-19.

The most notable merit of the current study is the investigation of the correlation between the number of COVID-19 cases and the volume of SP and its attributes. Sachini et al. (2021) inferred that the volume of European publications and COVID-19 cases were positively correlated. The current findings support this correlation in certain respects while denying it in others. Amazingly, the tests showed that variations in case volume have no effect on SP growth or the IC among MCs, whereas a positive relationship between this volume and the IC between MCs and the top ten countries was demonstrated. This was more evident for the IC between France, Italy, and Spain, which represent the highest-ranked MCs in terms of cases, and the United States, the United Kingdom, and Germany, which are all on the top ten list. These collaborative practices among the world's disease hotspots have previously been demonstrated by Sachini et al. (2021), Pal (2021), Belli et al. (2020), and Zhang et al. (2020). Among MCs, Italy was the most frequently mentioned as one of those spots.

In terms of the subjects, it was rational that the number of infections correlated positively with health and life sciences and COVID-19 papers under both the main subject areas and the majority of medical specialties. Surprisingly, there was a positive relationship with the social sciences, even though it appears to be less relevant to the pandemic context. This result seems to arise from calls from some researchers to investigate this crisis from a social perspective rather than just from a health perspective, especially with regard to the economic and behavioural implications (Usman et al., 2020; Zhang & Shaw, 2020). Further, the WHO launched a research roadmap in March 2020 in light of the outbreak, which includes some knowledge gaps classified under the social sciences (World Health Organization, 2020a).

The rise in the number of cases was also positively correlated with the increase in English publications, given that English is the global language of scientific research. During such a globally unprecedented situation, researchers from epidemic countries wished to communicate with one another urgently to exchange experiences and knowledge, which is harder to accomplish through other languages. The same interpretation can be extended to articles, reviews, and letters, positively associated with the growing number of infections, which require less time to edit, review, and publish, specifically during the COVID-19 period. Furthermore, the positive relationship between the number of cases and the increase in publishing in Q1, Q2, and Q3 journals demonstrated that the greater the number of cases was, the higher the motivation to achieve more qualified and accurate research as required by higher-impact journals.

## Conclusion

The current study makes a unique contribution by evaluating the COVID-19 impact on SP in all specialties focusing on the Mediterranean region, which was marked by diversity in the cumulative infection totals during the pandemic's first year. This evaluation also yielded unprecedented findings concerning the correlations between the volume and characteristics of SP and the case counts in the countries studied. Based on the results obtained, it can be said that the MCs witnessed a remarkable difference in the volume and features of SP during the COVID-19 outbreak, and this pandemic served as a powerful incentive for more international collaboration, conducting research on related topics, writing in the most common language, and using the fastest forms of communication. SP in MCs was notable not only for its expansion but also for its high-quality performance, as more publications appeared in prestigious journals. As such, this study can help academic leadership, policy-makers in higher education and research institutions, and funding agencies in those countries develop holistic visions and procedures that bridge identified research gaps and motivate researchers in all disciplines to continue generating research during this ongoing crisis.

## Limitations and future research

To ensure optimal coverage, the Scopes database and the SciVal tool were chosen to collect the required data. However, reliance on a single database can result in biased findings; subsequent comparative analyses could also use the Web of Science to accomplish the same objectives, and the comparisons may include preprint databases. The study did not rely on citation analysis as a criterion for research quality because it was expected that, due to the study's limited time span, the citations for this period, especially for 2020, would not reflect the real quality of the research. Rather, journal rankings were examined based on previous studies that used this benchmark (Oh & Kim, 2020) and confirmed the close relationship between these rankings and citations and that the most cited publications are usually published in journals with higher ranks (Sa’ed, 2016). Citations can be evaluated later after more time has passed. Furthermore, scientometric studies that explore the effect of a variable on scientific production usually cover two equal intervals, before and after the appearance of the variable, as in the study of (Ibrahim, 2018). In this paper, the collection of the required data started in January 2021. Therefore, the available research periods included one year before the emergence of COVID-19 (2019) and one year after it (2020). This interval can be extended to cover the following waves in future comparative work. Finally, the Mediterranean region was the sole focus of the study, and other regions could be analysed and compared.

### Electronic supplementary material

Below is the link to the electronic supplementary material.

Supplementary file1 (XLSX 143 KB)

## Appendix

See Tables 7 and 8.

**Table 7:** Scientific publishing before and during COVID-19 in Mediterranean countries according to the most COVID-19-related medical specialties

Regional rank	Continent /country	No. of cases	Virology		Infectious Diseases		Immunology		Microbiology		Parasitology		Tropical Medicine		Pharmacology & Pharmacy		Biochemistry & Molecular Biology		Medicine, Research & Experimental		Public, Environmental & Occupational Health	
			2019	2020	2019	2020	2019	2020	2019	2020	2019	2020	2019	2020	2019	2020	2019	2020	2019	2020	2019	2020
1	EU/France	2,576,420	13	503	5	165	6	207	1	30	1	1	0	2	0	25	1	9	2	28	0	82
2	EU/Italy	2,107,166	14	1159	8	443	2	164	1	54	0	1	0	0	0	22	1	27	1	56	1	202
3	EU/Spain	1,893,502	7	451	2	147	4	592	1	16	0	0	1	2	0	9	0	11	0	31	0	78
4	EU/Turkey	1,394,314	6	183	1	59	0	60	1	7	0	0	0	0	0	1	0	9	0	11	0	31
5	AF/Morocco	439,193	2	27	0	8	1	10	0	1	0	0	0	0	0	1	0	1	0	0	0	3
6	AS/Israel	408,277	0	96	0	51	0	44	0	1	0	0	0	0	0	1	0	0	0	5	0	12
7	EU/Croatia	212,007	1	13	0	9	0	4	0	0	0	0	0	0	0	0	0	0	0	4	0	7
8	AS/Lebanon	181,503	4	23	1	15	2	16	0	1	0	0	0	0	0	2	0	0	1	5	0	5
9	AS/Palestine	155,365	0	6	0	1	0	2	0	0	0	0	0	0	0	1	0	1	0	0	0	2
10	AF/Tunisia	139,140	3	11	2	3	2	3	0	0	0	0	0	0	0	0	0	1	1	1	0	1
11	EU/Greece	138,850	3	97	2	35	0	56	0	2	0	0	0	0	0	2	0	5	0	8	0	18
12	AF/Egypt	138,062	16	84	8	28	9	35	1	3	0	0	0	0	0	1	0	5	2	5	0	18
13	EU/Slovenia	123,950	0	15	0	6	0	2	0	1	0	0	0	0	0	1	0	1	0	1	0	5
14	EU/Bosnia and Herzegovina	110,985	0	2	0	0	0	0	0	0	0	0	0	0	0	0	0	0	0	0	0	1
15	AF/Libya	100,277	0	6	0	2	0	1	0	0	0	0	0	0	0	0	0	0	0	1	0	4
16	AF/Algeria	99,610	0	4	0	2	0	1	0	1	0	1	0	1	0	2	0	0	0	0	0	1
17	EU/Albania	58,316	0	2	0	0	0	0	0	0	0	0	0	0	0	0	0	0	0	1	0	0
18	EU/Montenegro	48,231	0	1	0	0	0	1	0	0	0	0	0	0	0	0	0	0	0	0	0	0
19	AS/Cyprus	22,651	0	12	0	6	0	5	0	1	0	0	0	0	0	0	0	0	0	0	0	2
20	EU/Malta	12,774	1	3	0	2	1	1	0	0	0	0	0	0	0	0	0	0	0	0	0	0
21	AS/Syria	11,434	0	1	0	1	0	0	0	0	0	0	0	0	0	0	0	0	0	0	0	0
22	EU/Gibraltar	1,973	0	0	0	0	0	0	0	0	0	0	0	0	0	0	0	0	0	0	0	0
23	EU/Monaco	875	0	0	0	0	0	0	0	0	0	0	0	0	0	0	0	0	0	0	0	0
Total		10,374,875	70	2699	29	983	27	1204	5	118	1	3	1	5	0	68	2	70	7	157	1	472

**Table 8:** Scientific publishing before and during COVID-19 in Mediterranean countries according to the publication type

Regional rank	Continent /country	No. of cases	Article		Conference paper		Review		Book chapter		Letter		Editorial	
			2019	2020	2019	2020	2019	2020	2019	2020	2019	2020	2019	2020
1	EU/France	2,576,420	81,948	91,047	18,901	11,872	8604	9795	3083	1791	2498	3284	2552	2608
2	EU/Italy	2,107,166	82,812	99,823	19,390	13,308	11,806	14,928	4319	2817	3263	5629	3045	3615
3	EU/Spain	1,893,502	76,567	89,473	9694	6687	7195	8901	2266	1274	1950	3046	1716	1874
4	EU/Turkey	1,394,314	39,372	48,679	5539	3522	1566	2093	1219	717	1041	1548	331	376
5	AF/Morocco	439,193	5390	6864	2606	1995	202	331	243	162	61	134	56	51
6	AS/Israel	408,277	16,613	19,416	2981	2021	1517	1707	798	383	355	434	335	367
7	EU/Croatia	212,007	5707	6182	1002	800	637	708	188	72	86	148	95	100
8	AS/Lebanon	181,503	2680	3337	691	391	291	542	83	52	63	100	43	77
9	AS/Palestine	155,365	771	1010	169	92	38	76	15	10	3	6	9	6
10	AF/Tunisia	139,140	5897	6624	1679	1274	212	259	173	72	118	98	45	33
11	EU/Greece	138,850	13,214	15,243	3588	2582	1846	2356	662	461	455	751	525	603
12	AF/Egypt	138,062	20,477	27,643	3161	1840	942	1419	544	223	175	308	103	132
13	EU/Slovenia	123,950	5108	5742	832	420	519	544	240	113	37	84	84	94
14	EU/Bosnia and Herzegovina	110,985	1026	1109	274	333	62	85	182	135	7	21	9	14
15	AF/Libya	100,277	371	465	139	165	20	40	16	12	3	16	4	1
16	AF/Algeria	99,610	5990	6729	2133	984	140	172	274	159	16	18	23	19
17	EU/Albania	58,316	361	495	106	52	37	36	35	29	4	7	3	11
18	EU/Montenegro	48,231	450	497	63	68	32	27	17	21	8	6	4	6
19	AS/Cyprus	22,651	2239	2854	577	445	204	340	92	63	44	61	78	86
20	EU/Malta	12,774	564	733	198	157	70	104	63	32	12	25	42	26
21	AS/Syria	11,434	469	632	47	38	35	42	8	11	9	11	0	0
22	EU/Gibraltar	1973	6	12	0	0	0	0	0	0	0	1	0	0
23	EU/Monaco	875	181	200	9	4	23	40	3	1	10	9	3	4
Total		10,374,875	3,68,213	4,34,809	73,779	49,050	35,998	44,545	14,523	8610	10,218	15,745	9105	10,103

Regional rank	Continent /country	No. of cases	Note		Erratum		Short survey		Book		Data paper		Retracted		Business article	
			2019	2020	2019	2020	2019	2020	2019	2020	2019	2020	2019	2020	2019	2020
1	EU/France	2,576,420	1265	1406	815	1021	1119	976	177	57	92	160	7	2	0	0
2	EU/Italy	2,107,166	1704	2197	824	1061	290	237	246	102	134	182	4	4	0	0
3	EU/Spain	1,893,502	982	1203	568	830	236	201	104	32	69	124	4	4	0	0
4	EU/Turkey	1,394,314	279	398	148	242	27	23	58	21	10	29	6	6	0	0
5	AF/Morocco	439,193	50	78	25	50	4	14	9	4	13	23	1	1	0	0
6	AS/Israel	408,277	312	347	171	224	65	37	104	26	16	8	1	0	0	1
7	EU/Croatia	212,007	69	87	34	36	18	14	8	6	2	10	1	0	0	0
8	AS/Lebanon	181,503	36	75	22	31	9	10	2	6	8	3	0	0	0	0
9	AS/Palestine	155,365	8	9	7	8	1	0	2	1	1	0	0	0	0	0
10	AF/Tunisia	139,140	46	61	33	52	12	13	5	0	6	4	3	1	0	0
11	EU/Greece	138,850	268	362	127	150	30	36	45	17	21	28	0	1	1	0
12	AF/Egypt	138,062	83	127	157	223	6	12	23	12	17	15	6	5	0	0
13	EU/Slovenia	123,950	46	47	38	58	18	3	9	3	11	12	2	0	0	0
14	EU/Bosnia and Herzegovina	110,985	4	9	3	8	0	1	2	0	0	0	0	1	0	0
15	AF/Libya	100,277	2	4	0	7	0	0	0	0	0	2	0	0	0	0
16	AF/Algeria	99,610	14	11	20	46	2	7	4	0	4	11	0	0	0	0
17	EU/Albania	58,316	1	0	0	5	0	0	3	0	0	0	0	0	0	0
18	EU/Montenegro	48,231	2	1	0	4	0	0	0	0	0	0	0	0	0	0
19	AS/Cyprus	22,651	30	41	14	25	1	5	5	6	2	6	0	0	0	0
20	EU/Malta	12,774	10	20	6	11	4	2	2	2	1	2	0	0	0	0
21	AS/Syria	11,434	3	1	4	7	0	2	0	0	0	2	0	0	0	0
22	EU/Gibraltar	1973	0	6	0	0	0	0	0	0	0	0	0	0	0	0
23	EU/Monaco	875	4	9	4	6	2	2	1	0	0	0	0	0	0	0
Total		10,374,875	5218	6499	3020	4105	1844	1595	809	295	407	621	35	25	1	1

## Footnotes

1 Prof. Syed Abdul Khaleque, Dr. Islam El Noby, Geographic Information Systems Dep., Helwan University.
